# From the neurobiological basis of comorbid alcohol dependence and depression to psychological treatment strategies: study protocol of a randomized controlled trial

**DOI:** 10.1186/s12888-017-1324-0

**Published:** 2017-04-28

**Authors:** Alena Becker, Anna M. Ehret, Peter Kirsch

**Affiliations:** Department of Clinical Psychology, Central Institute of Mental Health, Medical Faculty Mannheim/Heidelberg University, Mannheim, Germany

**Keywords:** Alcohol dependence, Depression, Reward processing, Mindfulness-based training, Behavioral Activation Training, Functional magnetic resonance imaging

## Abstract

**Background:**

Alcohol use disorder and depression occur commonly in the community. Even though this high-prevalence comorbidity is associated with poorer posttreatment outcomes and greater utilization of costly treatment services, existing treatment trials often exclude patients with comorbid depressive and alcohol use disorders. Past research suggests that symptoms such as craving and anhedonia might be associated with alterations within the reward circuit, while emotion regulation deficits are related to disruptions within the default mode network. The aim of this clinical neuroimaging study is to transfer previous research about the reward circuit and default mode network underlying alcohol use disorder and depression to achieve a better understanding of neural signatures characterizing their comorbidity. In addition, the neurobiological results will be used to test whether two psychotherapeutic intervention programs, mindfulness-based training and behavioral activation training, are able to positively influence the identified pathomechanisms.

**Methods:**

By means of functional magnetic resonance imaging (fMRI), 60 comorbid alcohol dependent and depressed patients are compared to 30 patients with depression only, 30 patients with alcohol use disorder only and 30 healthy control participants. Comorbid patients are randomized to either receive a behavioral activation or mindfulness based training and asked to participate in a second fMRI session and 3 month follow-up assessment. Thereby, we plan to explore whether these brief group psychotherapeutic intervention programs are able to positively influence the identified neurobiological pathomechanisms. The primary outcomes are reward and default mode network activity and connectivity evoked by paradigms measuring different facets of reward and emotion processing. Secondary outcome measures include craving and depression scores, as well as relapse rates. Predictors include participants’ characteristics, personality traits and indicators of mental health.

**Discussion:**

The objective of the project is to identify common and/or distinct neural signatures underlying the comorbidity of alcohol dependence and depression. If the neurobiological understanding of alcohol addiction and depression is improved, this could potentially serve as a key predictor of treatment response to specific types of behavioral or mindfulness therapies hypothesized to alter reward and resting state systems.

**Trial registration:**

German Clinical Trial Register DRKS00010249. The trial was registered January 23th 2017.

## Background

The prevalence of affective disorders in alcohol addiction is estimated to be 22.9% [1, 2] and the presence of alcohol dependence increases the risk for depression by a factor of almost 5 (odds ratio = 4.7) [3]. Despite the tremendous implications for prevention, treatment, and disease progression, the mechanisms underlying this comorbidity are not well understood. So far, studies with neuroimaging technologies, such as functional magnetic resonance imaging (fMRI), paired with behavioral measurement paradigms, have identified the brain’s reward system, as well as resting state networks like the default mode network (DMN) to play critical roles in the understanding of both alcohol dependence and depression.

## Target neurobiological mechanisms of alcohol dependence and depression: Reward System

Increasing evidence in humans and animals suggest that depression and alcohol dependence are associated with major disruptions within the reward circuit, which normally serves to guide our attention towards and consumption of natural rewards.

In major depression, several studies consistently report that activity in the nucleus accumbens (NAc), as part of the ventral striatum (VS), is reduced [4–7] reflecting a loss of reward function that drives symptoms such as anhedonia [8, 9]. Moreover, a recent study showed that depressive symptoms correlated with dysfunction in reward anticipation regardless of diagnostic entity [10]. Furthermore, specifically frontostriatal regions during reward processing have been suggested as an important psychophysiological marker of depression [11, 12]. In line with this, depressed individuals showed reduced frontostriatal activity during reward anticipation [5, 9, 13, 14] and outcome [13, 15].

In alcohol dependence, there is considerable evidence that the VS is activated by alcohol- associated cues and is thereby highly responsible for the incentive salience or “wanting” of goal objects such as alcohol [16–19]. However, whether the activation of the mesolimbic reward system results in alcohol consumption depends on the ability of higher-order structures to regulate incentive reward processing. Prevailing theories of addiction point to the involvement of the prefrontal cortex (PFC) not only through its engagement in executive functions, essential for goal-directed behavior (e.g. coding action-outcome conjunctions, salience attribution or self-control), but also through its monitoring of the mesolimbic reward system [20–22]. Past research suggested that aberrant incentive salience to rewards is not solely restricted to alcohol-associated cues, but also to non-dug rewards [23, 24]. These studies showed that alcohol dependent patients show reduced activation of the VS during the anticipation of monetary gain. On the contrary, recent results from our own group provided evidence that alcohol dependence is characterized by an imbalance between increased ventral striatal activation and weakened prefrontal control, as characterized by decreased frontostriatal connectivity during the anticipation of monetary reward [25]. These inconsistent findings might suggest the presence of different subgroups of alcohol dependent patients showing distinct forms of VS reward-regulating dysfunctions. Hence, it is hypothesized that alcohol dependent patients show diverse risk profiles characterizing distinct neural subgroups, which might be associated with comorbidities like depression.

In summary, in depression as well as in alcohol dependence, activity of the VS, as a pivotal node of the reward system, as well as frontostriatal connectivity have been shown to be altered during reward processing, emphasizing the essential importance of the reward system for both disorders.

## Target neurobiological mechanisms of alcohol dependence and depression: Default mode network

Recent efforts to understand the pathophysiology of major depression have focused on resting-state brain networks. There has been increased emphasis lately on conceptualizing depression as a disorder of functional brain connectivity [26] focusing on network interactions among cortical and subcortical regions. Among other identified neural networks, the default mode network (DMN) has received the most attention in the context of the clinical neuroscience of depression and has also sparked research interest in other psychiatric conditions [27]. The DMN is active and synchronized when the brain is “at rest” (i.e. not engaged in externally imposed goal-directed activity) and is compromised of the posterior cingulate cortex, medial prefrontal cortex and the angular gyri. The DMN has repeatedly been associated with rumination [28, 29], a recurrent, self-reflective and uncontrollable focus on depressed mood, which is thought to play a central aspect of the phenomenology of depression [30] and has also been found to be predictive for relapse and drinking status in alcohol dependent patients [31]. In a study from our own group, we could show that increased DMN connectivity in remitted depressed patients is associated with sadder mood, more rumination in daily life, as well as higher rumination and depression scores after six months [32].

A rather recent line of neuroimaging research has focused on resting state network properties in alcohol dependence. However, results are rather inconsistent, reporting either increased DMN connectivity [33] which was found to be related to relapse [34], mixed results of increased and reduced within DMN connectivity [35] or reduced connectivity [36] which was found to be correlated with poorer inhibitory control [37]. These heterogeneous results might implicate the existence of different subgroups or pathological states that modulates resting state connectivity in alcohol dependence. One modulating factor might be depression.

## Neurobiological informed treatment interventions for comorbidity: Psychotherapy

The ultimate goal of a better understanding of the neurobiological underpinnings is to develop preventive and more effective treatment strategies. Compared to antidepressant or anticraving medication, less research has examined functional neural responses after psychotherapy. Available data suggest that psychotherapy predicts metabolic changes in reward, cingulate and frontal cortices in alcohol dependence [38] and depression [39, 40].

To our knowledge, only a few studies evaluated the effects of a specific psychotherapeutic intervention like the brief Behavioral Activation Treatment (BAT) for depression on brain activation. Dichter and colleagues [41] report functional changes in reward associated structures including the dorsal striatum during reward anticipation after a BAT intervention but did not include a depressed control group. Looking on the DMN connectivity, Crowther and colleagues [42] found that the response to BAT in depressed patients was predicted by pretreatment connectivity of the right insula with the right middle temporal gyrus and the left intraparietal sulcus. Another psychotherapeutic intervention with potential effects on resting state networks is mindfulness based therapy (MBT). MBT is a meditation practice that is recently receiving growing attention for its potential clinical applications in the context of alcohol dependence and depression [43]. Neuroimaging studies showed that long-term meditators showed increased activation of prefrontal areas during meditation as compared with a rest condition or with matched control subjects naïve to meditation practice [44, 45].

In summary, dysfunctions in the reward circuit, as well as DMN have been identified as key domains underlying alcohol dependence and depression. We assume that these mechanisms also characterize the neurobiological underpinning of their comorbidity. Thereby, the identification of neural risk factors associated with their co-occurrence might help in understanding the pathophysiological mechanisms leading to their onset and course. The overall goal of improving our understanding of neurobiological mechanisms is to improve treatment. If the neurobiological understanding of alcohol dependence and depression is improved, this could potentially serve as a key predictor of treatment response to specific types of behavioral (BAT) or mindfulness (e.g. MBT) therapies shown to alter reward and resting state systems.

## Aim

The primary aim of this project is to identify neurobiological mechanisms underlying the psychopathology of comorbid alcohol dependence and depression. Subsequently, we plan to explore whether a brief psychotherapeutic intervention program is able to positively influence these pathomechanisms and if we can identify neurobiological signatures at baseline that predict the response to the particular intervention. To facilitate the achievement of the overall project objective, it has been broken down into several sub-objectives which represent a stepwise procedure towards the achievement of the primary aim:

1. Identification of neurobiological mechanisms underlying comorbid alcohol dependence and depression
  By means of comparing comorbid patients with alcohol dependent, depressed and healthy control subjects, we aim to understand distinct and common neuronal signatures and its target mechanisms that cut across both disorders.
  By identifying specific signatures related to the reward circuit and default mode network we will be able to recognize potential subgroups.
2. Development and application of psychotherapeutic interventions targeting these mechanisms
  If the neurobiological understanding of comorbid alcohol dependence and depression is improved, this could potentially serve as a key predictor of treatment response to specific established behavioral and mindfulness based intervention programs shown to improve clinical symptoms.
3. Evaluation of intervention on the neurobiological level
  By evaluating the psychotherapeutic interventions on the neurobiological level, we will be able to identify neurobiological markers of therapy efficiency (change of brain signatures after psychotherapeutic intervention).
4. Prediction of therapy response by means of neurobiological measures
  By predicting psychotherapeutic success by means of neurobiological signatures, we will gain first insights in neurobiological constructs to guide selection of distinct therapy targets and ultimately help to structure the personalization of psychotherapy.

## Methods

### Study design

**Fig. 1:**
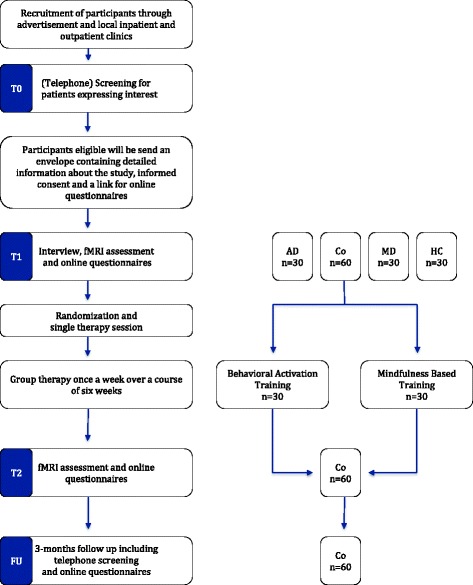
Flow Chart of the study design. Abbreviations: AUD = Alcohol use disorder patients, Co = Comorbid alcohol dependent and depressed patients, MD = Major depressive patients, HC = Healthy Controls, fMRI = functional magnetic resonance imaging

In this randomized-controlled trial, comorbid alcohol dependent and depressed patients will be compared to depressed only, alcohol dependent only and healthy control participants. All participants are screened and after examination of inclusion and exclusion criteria admitted to the study. All participants will receive a baseline fMRI session (T1). Subsequently, only comorbid alcohol dependent and depressed patients will be randomized to either receiving group BAT or MBT training for 6 weeks. Afterwards comorbid patients will receive an outcome fMRI session (T2) and will be contacted after three months for a catamnestic exploration. For a flow chart of the study design please refer to Fig. 1.

### Participants

Sixty comorbid alcohol dependent and depressed patients, 30 alcohol dependent only and 30 depressed only patients will be included for T1 assessment. Further, 30 healthy control participants (matched according to age, gender and educational level) will be recruited.

### Eligibility

All participants will be 1) aged between 18 and 65 years, 2) have to provide informed consent, 3) be right-handed, 4) show an appropriate comprehension of the German language and 5) must be able to accurately watch visual stimuli in the scanner environment. Alcohol use disorder and depression will be diagnosed according to DSM-5 criteria and patients must be abstinent for at least 5 days before scanning. Exclusion criteria for all participants include 1) psychiatric Axis I disorders as measured by the SCID I interview within the past 12-months (except for patients alcohol and nicotine abuse and depressive symptoms), 2) neurological disorders, 3) MRI specific contraindications such as having a pace maker or implanted metal, 4) positive drug screening (opioids, cannabinoids, benzodiazepines, barbiturates, cocaine, amphetamines), 5) any current psychoactive medication except anti-depressants, 6) pregnancy and 7) suicidal tendencies. During the participation in the group therapies, comorbid participants should not enroll in any other CBT or individual psychotherapeutic treatment.

### Sample size

Power calculations were conducted by means of the G*Power 3.1 software package [46]. According to pilot studies, an effect size of at least d = 1 can be assumed for the different paradigms and comparisons. For region of interest analysis with regions of 200 voxels, a sample of 30 per group should be sufficient to reach a power of 80% to detect differences at *p* < .05 (Bonferroni corrected for multiple tests). For the intervention study the same comorbid alcohol dependent and depressed participants will be randomly assigned to one of two group therapy interventions and asked to participate in a second fMRI session and follow up measurement. When assuming a small to medium effect size (*f* = .2) for the group x time interaction and a correlation among repeated measures of.7 (as typical for fMRI data), we achieve a power of 80% to detect differences at *p* < .05 (Boferroni corrected for a k = 200, region of interest) on the neural level even when we have to face a drop-out rate of 10%. While we expect an attrition rate of 20%, recruitment will be continued until the desired sample sizes are reached and drop-outs will be replaced by new participants.

### Sample recruitment

Participants of the study are recruited by means of several sources in the community around Mannheim, Germany. These include advertisement in local newspapers, announcements on our institutes’ homepage, flyers in local businesses and university campuses; and contact with potential referral sources, for example general practitioners, private psychologists and psychiatrists, as well as local psychiatric inpatient and outpatient units.

Prospective participants express their interest to the research team through email or phone call. After carefully informing the participants about the aim, content, duration and potential risks and benefits of the study, trained research assistants assess initial eligibility using a specifically developed telephone screening. In case the participants are currently in one of the local inpatient or outpatient facilities of the institute, participants will be personally approached and screened. If the individual meets the inclusion criteria, they receive a letter including a detailed study description, the informed consent, time and date of their study appointment and an online link with questionnaires.

### Study procedure

Before participants will be finally included in the study, any open questions, risks and benefits will be discussed with a trained psychologist at the T1 assessment. On agreement of the participant, the informed consent form will be signed and a copy handed to the participant. Afterwards, participants will undergo a psychological interview assessing previously received therapy, current medication and for patients symptoms of depression or alcohol use disorder according to DSM-5. Before the fMRI measurement starts, all paradigms are explained to the participant. Also before and after the fMRI session, participants are asked to fill out questionnaires regarding their current mood and craving. Only comorbid alcohol dependent and depressed patients will be randomized to either receive BAT or MBT group training for 6 weeks. For comorbid patients, the fMRI procedure followed at T1 will be repeated at T2. Afterwards, comorbid patients will be contacted after three months for a catamnestic exploration. All participants will be compensated for their time with 15€ per scanning session plus the money won in the reward paradigm. To promote participant retention and complete follow-up we will contact the comorbid patients by phone during the follow-up period.

### Randomization

After completion of baseline assessment, comorbid patients will be randomly assigned to one of the two group interventions. To reduce the time between the initial study recruitment and the start of the group therapy, the randomization order will be determined with a block randomization with 6 participants per block for each treatment arm. Randomization will be performed by a researcher who is not involved in the recruitment, using a computerized randomization tool implemented in MATLAB (The MathWorks Inc., Natick, Massachusetts, United States). Participants will be recruited and enrolled by trained research staff; the allocation sequence will be concealed from them. Since the study offers psychotherapeutic interventions, patients are not blinded to their intervention arms.

### Psychotherapeutic Interventions

Comorbid patients will be randomly assigned to one of two group therapies consisting of 6 participants. Both interventions consist of six sessions of 2 h therapy with 1 session per week offered by a psychotherapist. To increase adherence, ﻿each participant will receive an individual therapy session before the group therapy begins. The group therapy protocols in both arms are manualized and supervised by an experienced psychotherapist to ensure protocol adherence. Patients who missed more than one session or experienced a severe relapse during the intervention period will be excluded from the protocol. In the unexpected case of an adverse event or harm during the psychotherapeutic intervention (for example, severe aggravation like suicidality), the affected patient will also be excluded and additional in- or outpatient treatment will be offered by the psychotherapist.

#### Behavioral activation treatment

Behavioral Activation Treatment (BAT) is a structured and validated psychotherapy intervention designed to increase engagement with functional, potentially rewarding behaviors [47]. Treatment proceeds through a series of structured units that (a) provide psychoeducation about depression and alcohol dependence, as well as a rationale for the treatment approach, (b) assess and monitor baseline activity levels, (c) identify and support more functional behaviors and personally valued goals as an alternative strategy to cope with cravings and low mood, (d) monitor, support, and reward achieving behavioral goals, and (e) improve problem solving skills. Previous studies have demonstrated that BAT for depression effectively reduces depressive symptoms and is well tolerated in outpatient settings [48].

#### Mindfulness based therapy

The mindfulness based intervention aims to increase awareness for and help patients to accept situational cues, negative thoughts, feelings, and behavioral impulses that have been associated with drinking [49, 50]. Participants are guided through structured units that (a) provide psychoeducation about depression and alcohol dependence, as well as a rationale for the treatment approach, (b) increase patients’ awareness of different experiences including low mood and cravings, (c) help clients to distance themselves from challenging experiences, e.g. by focusing on their breathing, (d) help them to respond to pleasant and unpleasant sensations with acceptance, and (e) foster their sense of strength and self-efficacy by enduring negative experiences and withstanding behavioral impulses.

### Additional information

The study protocol is in accordance with the Standard Protocol Items: Recommendations for Interventional Trials (SPIRIT) guidelines for reporting interventional trials. Ethical approval and consent has been obtained by the leading ethics committee of the Medical Faculty Mannheim/University of Heidelberg (reference number: 2015-606 N-MA). The study will be conducted in compliance with the guidelines for Good Clinical Practice and the Declaration of Helsinki. Participants will provide informed consent prior to enrolment in the study. The trial is registered in the German Clinical Trial Registration (DRKS00010249). Any changes to the protocol will be described in this trial registry.

The principal investigator (PK), independent from the sponsor and with no competing interests to report, is responsible for data monitoring. All adverse events are documented. Interim analyzes are not planned but cross-sectional comparisons for T1 data will be conducted after inclusion of all T1 participants and before data collections at T2 and FU are completed. The day-to-day management of the study (e.g. contact with the participants and the invitation to conduct online surveys) is carried out by advanced researchers. The publication of the results will be independent of the results of the study, even if the intervention proves to have no influence on brain activation and connectivity. Authorship eligibility will be decided according to the guidelines for safeguarding good scientific practice provided be the German Research Association [51]. A written summary of the results will also be made available to the participants after completion of the study. Data, statistical parameters and statistical code of the study will be made accessible to interested researchers after request. Due to data safety concerns, raw data cannot made openly accessible for the public.

### fMRI assessment battery

#### Reward paradigm

In this event-related paradigm, participants are asked to respond to a flashlight as fast as possible by pressing a button. Preceding the flashlight, an arrow cue informs the participant about the rewarding consequences of their response to the flash stimulus. Four conditions are included in the paradigm: (1) in the so called monetary reward condition (arrow up) participants’ fast response to the flash is rewarded with 2€; (2) in the punishment avoidance condition (arrow down) a slow response is punished by a loss of 2€ while a fast response is rewarded by the avoidance of that loss; (3) the verbal feedback condition (vertical double arrow) allows the participants to receive verbal feedback on their performance; (4) in the passive control condition (horizontal double arrow) no response is required. During the outcome phase, the participants see the reward of the latest trial (“2€”, “0″ or “Fast response!”, “Too slow”) as well as their cumulative total gain at that point in time. In order to ensure that all participants are able to win some money and have comparable winnings, the threshold for a fast response is adaptively tailored for each trial to the individual response times of the subject. The inter-trial interval was randomly varied between 6 and 9 s. Each condition is presented 10 times in a pseudo-randomized trial order with no more than two equal conditions in succession. The total run length is 9 min. The monetary reward anticipation task [52] has been shown to reliably and robustly activate the ventral striatum including the nucleus accumbens [53].

#### Erotic incentive delay task

In order to measure different categories of reward processing, a variant of our own monetary reward task [52] comprising erotic stimuli will be performed. Similar to the description above, participants have to respond to a cued probe to receive either erotic stimuli or matched social control stimuli. Again during the anticipation phase, distinct cues preceding the target inform the participants whether a rewarding erotic image will be shown or not. In previous studies it has been shown that striatal reward-associated brain activations can be reliably observed when erotic pictures are presented [54].

#### Resting state/mood induction paradigm

The so called mood induction paradigm has been successfully implemented in different studies [32]. Here, we will use a modified version of the paradigm in order to directly test intervention associated activation and connectivity patterns. The paradigm consists of five blocks, each of which lasts 4.5 min: a resting state block, a sad mood induction block, one alcohol cue-reactivity block, one mindfulness-based block and one behavioral activation block. The mindfulness-based block and the behavioral activation block are counterbalanced.

During the resting state phase, participants are told to keep their eyes open and background pink noise is presented. During the sad mood induction phase, key words to remind participants of personal negative life events and background sad music (excerpt from Albinoni/Giazotto, Adagio in g-minor) are presented. Participants are asked to concentrate on the negative life events and empathize with the situation. During the alcohol cue-reactivity phase, participants are presented images of either beer or wine (depending on the participants preference) while they receive corresponding audio induction statements instructing them to imagine alcohol-associated situations. In the mindfulness- based phase, again, participants are presented images of beer or wine while this time they listen to mindfulness-based audio instructions. Correspondingly, in the behavioral activation training phase, participants are played audio instructions associated with imagining activities while viewing alcohol-associated pictures. In each phase, 9 alcohol-associated cues are presented for 15 s, while simultaneously 9 audio statements are played. After each picture a fixation cross is presented for also 15 s. After each block, participants are instructed to rate their mood by means of the Positive and Negative Affect Schedule (PANAS), as well as their craving and self-control to abstain. For the T2 assessment, a different set of alcohol-associated pictures is presented.

As mentioned above, the mindfulness-based block and the behavioral activation block are counterbalanced. Thus, a second randomization list will ensure that at T1 the order of the experimental blocks will be equally distributed above all participants. Comorbid patients will receive the reverse order of their T1 assessment at the T2 fMRI scanning.

### Anatomical image

In order to assess the individual brain morphology of each participant, high-resolution T1-weighted anatomical images (MPRAGE) will be collected.

### Primary outcome

In this study, neural activation and connectivity in the reward system and default mode network are the primary outcome measures. Neural correlates of reward and emotion processing in patients and healthy controls will be assessed by means of fMRI. While the first measurement will identify common and/or distinct neural signatures underlying the comorbidity of alcohol dependence and depression, the second measurement allows investigating the influence of the psychotherapeutic interventions on these target brain mechanisms.

### Secondary outcome measures

Secondary outcome measures include craving and depression scores, as well as relapse rates.

### Predictors

Predictors include participants’ characteristics and personality traits as assessed by questionnaires (See Table 1), as well as ratings before, during and after scanning.

**Table 1:** Schedule of measurements

Variable	Instrument	T0	T1	T2	T3
Neural activation and connectivity	fMRI assessment battery		⨯	⨯	
Demographic information	Interview/Self-report	⨯			
Psychopathology	Structured Clinical Interview for DSM-IV, Axis I,II	⨯			
Medication	Self-report		⨯		
Health Care resource utilisation	Interview/Self-report		⨯		
Alcohol Use	Form 90 Interview		⨯		⨯
Alcohol Dependence	Alcohol Dependence Scale		⨯	⨯	⨯
Craving	Obsessive Compulsive Drinking Scale Alcohol Urge Questionnaire		⨯	⨯	⨯
Depression	Beck Depression Inventar Montgomery and Asperg Depression Rating Scale		⨯	⨯	⨯
Rumination	Response-Styles-Questionnaire Perseverative Thinking Questionnaire		⨯	⨯	⨯
Mindfulness	Mindfulness Attention and Awareness Scale		⨯	⨯	⨯
Quality of life	Münchner Lebensqualitäts-Dimensionen Liste		⨯	⨯	⨯
Self-confidence	Rosenberg Selbstwert Skala Self-Acceptance Scale Forms of Self-Criticizing/Attacking and Self- Reassuring Scale		⨯	⨯	⨯
Smoking	Fargerstrom Test for Nicotine Dependence		⨯	⨯	⨯
Intervention Satisfaction	Self-report			⨯	

### Data analyses plan

Analyses will be conducted on an intention to treat and treatment completers principle.

### Behavioral data analyses

Analysis will be completed by using (repeated measures) analysis of variance (AN(C)OVA) in order to test group differences (AUD vs MD vs Co vs HC) and treatment groups (BAT vs MBT). Difference scores will be calculated. All measures will be compared between groups, as well as treatment groups and interaction effects will be explored. Wherever appropriate, missing data will be imputed using multiple imputation.

### Activation fMRI analysis

Preprocessing as well as statistical analyses of the experimental fMRI paradigms will be performed using SPM 12 (Wellcome Department of Cognitive Neurology, London, UK). In order to reduce systematic motion-related artifacts known to be especially prevalent in patient-control studies [55], we use the ART toolbox (http://www.nitrc.org/projects/artifact_detect) to detect outlier volumes. The ART software identifies outliers if the normalized z-score of the global mean intensity of a volume exceed a specified threshold and/or if extensive composite movement from a preceding image occurs. Preprocessing will be conducted with the standard procedure analysis including slice time correction, realignment, co-registration, normalizing, smoothing. Statistical analysis on the first (individual) level will be performed by modeling the different conditions using boxcar functions convoluted with the hemodynamic response function as explanatory variables within the context of the general linear model (GLM) on a voxel-by-voxel basis.

### Connectivity fMRI analysis

Task and resting state fMRI data will additionally be analyzed by connectivity approaches. To account for physiological and movement artifacts which strongly introduce artificial correlations into the data, heart beat and respiration will be monitored during scanning and physiological correction will be conducted with the software Aztec. Afterwards, images are preprocessed as described before by SPM 12. Task-dependent connectivity will be examined by functional connectivity and psychophysiological interaction analyses. Resting state data will be examined by conventional as well as dynamic functional connectivity analysis. Analyses in both task and rest will be conducted in a hypothesis-driven network-specific as well as more exploratory whole-brain manner [56]. The whole-brain results will be further analyzed by graph theoretical tools to elucidate relevant changes in functional network topology.

### Data management

The project data will be saved on the central server of the institute. The IT department professionally maintains the server; thereby a daily backup of all data is performed. Additionally, a network independent backup will be saved on local hard drive. Experimental data, as well as questionnaires will be saved in a pseudonymized form, separately from consent forms that contain the names of the participants. In addition, hard copy storage of reports, protocols, presentations or publication drafts will be realized. All data will be pseudonymized using unique study codes, which will be used to code and file all electronic information. The key linking this code to participant identity will be stored in a secured file, access to this key is available only to designated members of the research team. These researchers will have unrestricted access to all study data. As mentioned above, anonymized data will be made available for re- or meta-analysis after request.

## Discussion

Although there is significant knowledge of the environmental and neurobiological factors involved in depression and alcohol dependence, only a few studies have translated these empirical findings into a better understanding and treatment of comorbid patients. The planned project is set-up to transfer previous research about the reward circuit and default mode network underlying alcohol dependence and depression to achieve a better understanding of neural signatures characterizing their comorbidity. By means of diverse experimental paradigms allowing a comprehensive characterization of reward and default mode network activity and connectivity, we are planning to assess distinct and common mechanisms underlying comorbid alcohol and depressed patients. In addition, the neurobiological results will be used to test whether a brief psychotherapeutic intervention program is able to positively influence the identified pathomechanisms. By gaining a better understanding of diverse phenotypes and their underlying neurobiological signatures, it might be possible to improve clinical outcomes by offering specific types of psychotherapy. Furthermore, we should find support for our hypotheses that established behavioral and mindfulness based intervention programs specifically alter pathological neurobiological functions. By this, we will be able to identify neurobiological markers of therapy efficiency. The prediction of psychotherapeutic success based on neurobiological signatures will help to guide the selection of specific therapeutic options. Ultimately, by means of identifying and validating the use of objective, biological indicators associated with core mechanisms of mental disorders, advances in personalized medicine may be achieved.

One limitation of the study might concern the lack of a proper control group for the investigation of the treatment effects (for example waiting control group). However, this study was not designed to evaluate treatment effects per se but rather to investigate the neurobiological correlates of established psychotherapeutic interventions. With this, we are specifically interested if the targeted interventions positively alter the pathophysiology of comorbid patients and whether this is restricted to patients with a specific neurobiological signature. A second challenge affects the recruitment of comorbid depressed and alcohol dependent patients. Not only for the fMRI measurement, but also for the feasibility of the group therapy patients abstinence is a prerequisite. In order to motivate comorbid patients to participate in the group therapy and to stay abstinent, we want to guarantee a fast study enrolment, as well as ensure a short latency between study enrolment and begin of group therapy. For that reason, we decided for a group randomization of 6 participants per block. With this, we might risk recruitment-dependent biases. However, due to many different recruitment strategies and sources, we hope to reduce this potential bias to a minimum.

To reach the project results is one of the key issues and core challenges to contribute to a better neurobiological understanding and treatment of comorbid depression and alcohol dependence. The project will help to improve knowledge on pathophysiology, identify targets for treatment development, detect subgroups for treatment selection, and facilitate the use of neurobiology findings in the understanding of comorbid alcohol dependence and depression.

## Abbreviations

AUD: Alcohol Use Disorder
BAT: Behavioral Activation Training
Co: Comorbid patients
DMN: Default mode network
fMRI: Functional magnetic resonance imaging
HC: Healthy controls
MBT: Minfulness based training
MD: Major depression
NAc: Nucleus Accumbens
PFC: Prefrontal Cortex
VS: Ventral Striatum
